# Genome-wide identification of *Bacillus subtilis* Zur-binding sites associated with a Zur box expands its known regulatory network

**DOI:** 10.1186/s12866-015-0345-4

**Published:** 2015-02-04

**Authors:** Eric Prestel, Philippe Noirot, Sandrine Auger

**Affiliations:** INRA, UMR1319 Micalis, F-78352 Jouy-en-Josas, France; AgroParisTech, UMR Micalis, F-78352 Jouy-en-Josas, France

**Keywords:** Zur regulator, *B. subtilis*, ChIP-on-chip, Zinc homeostasis, Disulfide stress

## Abstract

**Background:**

The *Bacillus subtilis* Zur transcription factor recognizes a specific DNA motif, the Zur box, to repress expression of genes in response to zinc availability. Although several Zur-regulated genes are well characterized, a genome-wide mapping of Zur-binding sites is needed to define further the set of genes directly regulated by this protein.

**Results:**

Using chromatin immunoprecipitation coupled with hybridization to DNA tiling arrays (ChIP-on-chip), we reported the identification of 80 inter- and intragenic chromosomal sites bound by Zur. Seven Zur-binding regions constitute the Zur primary regulon while 35 newly identified targets were associated with a predicted Zur box. Using transcriptional fusions an intragenic Zur box was showed to promote a full Zur-mediated repression when placed within a promoter region. In addition, intragenic Zur boxes appeared to mediate a transcriptional *cis*-repressive effect (4- to 9-fold) but the function of Zur at these sites remains unclear. Zur binding to intragenic Zur boxes could prime an intricate mechanisms of regulation of the transcription elongation, possibly with other transcriptional factors. However, the disruption of zinc homeostasis in Δ*zur* cells likely affects many cellular processes masking direct Zur-dependent effects. Finally, most Zur-binding sites were located near or within genes responsive to disulfide stress. These findings expand the potential Zur regulon and reveal unknown interconnections between zinc and redox homeostasis regulatory networks.

**Conclusions:**

Our findings considerably expand the potential Zur regulon, and reveal a new level of complexity in Zur binding to its targets via a Zur box motif and via a yet unknown mechanism that remains to be characterized.

**Electronic supplementary material:**

The online version of this article (doi:10.1186/s12866-015-0345-4) contains supplementary material, which is available to authorized users.

## Background

Zinc is an essential trace element for all forms of life. It serves as structural scaffold for protein folding and as cofactor for many enzymes and DNA-binding proteins [1,2]. However, due to its toxicity, mechanisms for zinc acquisition and efflux are tightly regulated according to metal ion requirements [3-5].

In the Gram positive bacterium *Bacillus subtilis*, transcription of genes involved in zinc homeostasis is regulated by Zur, a metalloprotein that binds Zn(II) as corepressor [6-9]. *In vivo*, Zur forms a homodimer that binds to a conserved DNA motif, the Zur box, which overlaps the σ^A^-type promoter elements in target genes [10]. DNA-binding studies demonstrated that Zur requires a minimal 9-1-9 inverted repeat motif for high-affinity binding [11]. A stepwise activation model predicts that Zur may respond to a wide range of intracellular Zn(II) concentrations to gradually repress the Zur regulon [12]. Zur represses expression of genes encoding a high-affinity zinc ABC transporter ZnuACB [7], a putative low affinity zinc uptake system YciBC [10], a GTP cyclohydrolase IB involved in folate biosynthesis FolEB [13], and zinc-independent alternative ribosomal proteins (RpmEB, RpmGC, and RpsNB) [14-18]. In *B. subtilis*, zinc is also imported by the P-type ATPase ZosA, whose expression is controlled by the peroxide-sensing regulator, PerR [19]. Induction of ZosA in response to hydrogen peroxide stress leads to Zn(II) uptake, which plays an important protective role against oxidative stress damage [19]. Both ZosA and ZnuACB zinc transporters are involved in the competence developmental process [20]. Zinc homeostasis is also maintained in *B. subtilis* thanks to a zinc-inducible efflux pump CzcD important for growth in the presence of high concentration of Zn(II) [5,21]. Expression of this system is regulated at the transcriptional level by the metalloregulator CzrA [5].

Despite knowledge of Zur-mediated regulation of zinc homeostasis, a global identification of the genes directly under Zur control is still missing. Here, we used chromatin immunoprecipitation of Zur-DNA complexes coupled with hybridization of DNA to tiled oligonucleotides arrays (ChIP-on-chip) to identify regions enriched for Zur DNA-binding sites *in vivo*, at the genomic scale. We provide evidence that Zur binds to 80 regions on the chromosome, including previously known promoter regions of the Zur primary regulon as well as a number of inter- and intragenic regions. Half of the newly identified binding sites is associated with a predicted Zur box. We showed that an intragenic Zur box was functional to mediate a Zur-dependent repression when inserted in a promoter region. In addition, several intragenic Zur boxes were able to act as transcriptional *cis*-repressive element but the direct role of Zur at intragenic sites remains unclear. Finally, our study suggests that Zur binding to newly identified targets could be primed to fine-tune gene expression in interplay with other transcription factors in response to specific conditions such as the disulfide stress.

## Results

### C-terminally SPA-tagged Zur is a functional regulator

The *B. subtilis* chromosome was modified at the *zur* locus to express Zur fused at its C-terminus with the SPA tag (Zur^SPA^). In the resulting *zur*::*zur*-*spa* strain, the Zur^SPA^ protein is under the control of its native expression signals. To examine the activity of the Zur^SPA^ fusion protein, the *yciC* promoter region was fused with the *lacZ* reporter gene and introduced at the *amyE* locus in wild-type, *zur*::*zur*-*spa* and Δ*zur*::*aphA3* strains (Methods) (Table 1). Expression of *yciC*, monitored by measuring the β-galactosidase activities, was repressed in wild-type and *zur*::*zur*-*spa* cells (β-galactosidase activity ≤ 5 UE) whereas it was increased by a 160-fold factor in Δ*zur* cells. Thus, Zur^SPA^ functionally repressed *yciC* expression as wild-type Zur.

**Table 1:** ***B. subtilis*** **strains used in this study**

**Strain**	**Genotype** ^**a**^	**Source**
BSB1	*trp* ^*+*^	(Nicolas *et al*., [26])
BSAS36	*zur::zur-spa erm*	This work
BSAS39	*amyE*::p*yciC*’-*lacZ cat*	This work
BSAS44	*amyE*::p*yciC*’-*lacZ cat zur-*spa *erm*	This work
BSAS45	Δ*zur*::*aphA3*	This work
BSAS49	*amyE*::p*yciC*’-*lacZ cat* Δ*zur*::*aphA3*	This work
BSAS225	*amyE*::pA*ymaD*’-*lacZ cat*	This work
BSAS331	*amyE*::pB*ymaD*’-*lacZ cat*	This work
BSAS227	*amyE*::pA*ydeO*’-*lacZ cat*	This work
BSAS332	*amyE*::pB*ydeO*’-*lacZ cat*	This work
BSAS229	*amyE*::pA*ywhC*’-*lacZ cat*	This work
BSAS333	*amyE*::pB*ywhC*’-*lacZ cat*	This work
BSAS239	Δ*ymaD*::*aphA3*	This work
BSAS240	*amyE*::pA*ktrD*’-*lacZ cat*	This work
BSAS329	*amyE*::pB*ktrD*’-*lacZ cat*	This work
BSAS249	*amyE*::pA*yrpE*’-*lacZ cat*	This work
BSAS250	*amyE*::pA*yrpE*’-*lacZ cat* Δ*zur*::*aphA3*	This work
BSAS251	*amyE*::pB*yrpE*’-*lacZ cat*	This work
BSAS252	*amyE*::pB*yrpE*’-*lacZ cat* Δ*zur*::*aphA3*	This work
BSAS296	Δ*spx*::*spc*	This work
BLUC201	*amyE*::pA*ymaD*'-*luc cat*	This work
BLUC204	*amyE*::pA*ymaD*'-*luc cat* Δ*spx*::*spc*	This work
BLUC205	*amyE*::pA*ymaD*'-*luc cat* Δ*zur*::*aphA3*	This work
BLUC202	*amyE*::pB*ymaD*'-*luc cat*	This work
BLUC205	*amyE*::pB*ymaD*'-*luc cat* Δ*zur*::*aphA3*	This work
BLUC227	*amyE*::pB*ymaD*’-*lacZ cat* Δ*spx*::*spc*	This work

^a^ *cat,* pC194 chloramphenicol acetyl-transferase gene; *aphA3*, *Enterococcus faecalis* kanamycin-resistance gene; *erm*, erythromycin-resistance gene; *spc*, *Staphylococcus aureus* spectinomycin-resistance gene.

We further tested the effect of ion starvation on *yciC*'-*lacZ* expression in the same genetic backgrounds. The strains were cultivated in MS medium and samples were spread onto solid medium containing X-gal. A drop of 100 μM EDTA, an ion chelating agent, was deposited at the center of the plates. After incubation, a characteristic blue ring was observed around the EDTA drop (Additional file 1: Figure S1) indicating that ion deficiency induced *yciC* expression in cells synthesizing Zur as well as Zur^SPA^ proteins. The binding of Zur^SPA^ to the *yciC* promoter region was reversible upon metal ion starvation. We concluded from this data that the Zur^SPA^ fusion protein was functional for transcriptional regulation.

### Genome-wide mapping of Zur binding sites

**Figure 1:**
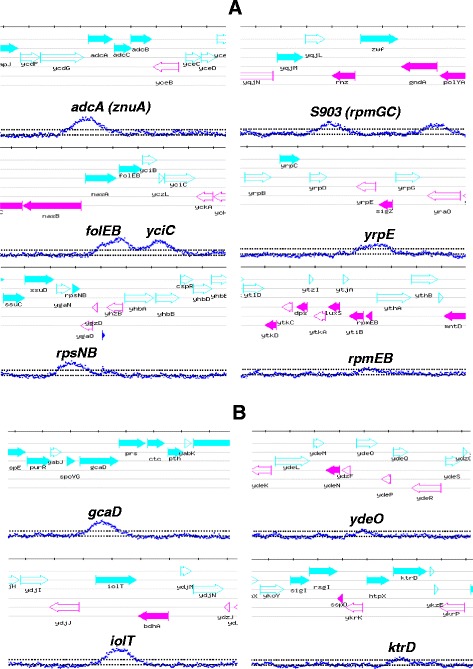
**Typical Zur-binding sites during growth of** ***B. subtilis*** **in exponential phase in LB medium.** Each of the 10 panels represents a chromosomal region with annotated genes. The ChIP-on-chip profiles with the peaks detected are represented in blue. **(A)** Zur^SPA^-binding sites in promoter regions of the known Zur regulon. **(B)** Zur-binding sites in intragenic regions.

To identify Zur-binding targets in the *B. subtilis* genome, we carried out ChIP-on-chip experiments. The *zur*::*zur*-*spa* strain was grown in LB medium to exponential phase. After cross-linking, Zur-bound DNA was immunoprecipitated using a SPA tag specific antibody. The signals from two independent experiments were processed and peaks corresponding to Zur^SPA^ binding sites were selected using a cut-off value of 4.0 (see Methods). Overall 80 enriched DNA regions were identified from the ChIP-on-chip signals (Additional file 2: Table S1). Among them, 5 were located in genomic regions known to belong to the Zur regulon (Figure 1A) [22]. As in parallel, we used the ChIP-on-chip methodology to study various *B. subtilis* transcription factors [23,24], we observed that all the peaks detected with Zur were exclusively specific to this regulator.

We further compared our data with previous *in silico* studies [25]. The promoter-proximal Zur-binding sites upstream of *yrpE* and the pseudogene *S903* (also named *rpmGC*) exhibited Zur box motifs. We then tested expression of the *yrpE* gene in wild-type and Δ*zur* cells by using a transcriptional fusion between the *yrpE* promoter region and the *lacZ* reporter gene. Expression of the P*yrpE* promoter was 30-fold increased in a *zur* mutant compared to the wild-type during the exponential phase of growth (Additional file 1: Figure S1). These results demonstrated the Zur-dependent regulation of *yrpE*. We also detected Zur binding upstream of the short *yczL* gene, encoding a protein of unknown function. Expression of *yczL* is co-regulated with *yciC* and is driven from a single promoter leading to the *yczLyciC* transcript [26]. Altogether, these findings delineated the Zur primary regulon, which is now composed of 11 genes expressed from 7 distinct promoters (*znuABC*, *folEByciB*, *rpsNB*, *rpmEB*, *rpmGC*, *yczLyciC* and *yrpE*) fulfilling three criteria: (i) *in vivo* Zur binding in ChIP-on-chip experiments; (ii) presence of a Zur box; (iii) Zur-dependent regulation of expression.

In addition, 37 additional promoter-proximal Zur binding sites were detected less than 200 base-pairs upstream of a translational start site (Additional file 2: Table S1) suggesting a Zur-dependent expression and existence of new candidates to be part of the Zur regulon. The expression of all the genes belonging to the Zur primary regulon displayed a similar expression profile whereas the expression of the genes closed to the 37 newly identified promoter-proximal sites did not appear correlated with the Zur primary regulon [26]. These genes might be controlled by Zur under specific unknown conditions. The presence of predicted Zur boxes in these regions is discussed below.

Finally, 35 peaks were located within intragenic regions more than 200 bp downstream a start codon (Figure 1B). The location of these sites was intriguing since none Zur intragenic binding site has so far been described.

### Prediction of Zur boxes within Zur-binding sites

**Figure 2:**
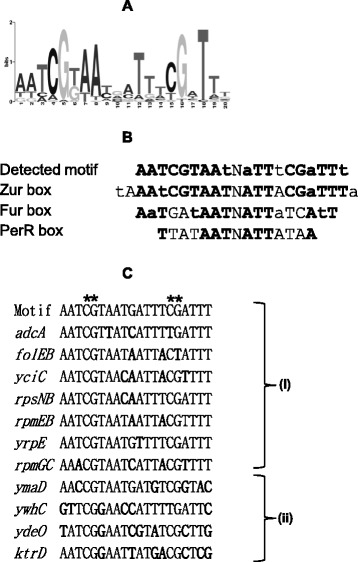
**Zur box motifs present in Zur-binding sites identified in ChIP-on-chip experiments. (A)** The 20 nt palindromic consensus sequences identified from the *in vivo* Zur binding sites. The size of the nucleotide at each position indicates its relative prevalence in sequences used as training set for MEME algorithm [30]. **(B)** Alignment of the motif identified in this work with the previously reported Zur box, Fur box and PerR box. Common nucleotides are indicated in bold. **(C)** Alignment of Zur box sequences: (i) Zur boxes in promoter regions of genes belonging to the Zur regulon; (ii) intragenic Zur boxes in Zur binding regions detected by ChIP-on-chip. Nucleotides matching the consensus (plain) and nucleotides different from the consensus (bold) are indicated. Stars mark the critical positions for Zur binding as previously described [11].

To investigate the presence of Zur boxes within the newly identified Zur-binding sites, we performed *in silico* analyses. Zur binds to DNA sites, which display high sequence similarity to those recognized by Fur and PerR although regulon overlap in naturally occurring promoter/operator sites has not been reported [27-29]. Here, we used the MEME standard bioinformatic method [30] to identify common motifs among genomic regions representing 100 bp centered at each Zur-binding sites. An appealing feature of this program is its ability to automatically compute optimal motif widths, in contrast to the majority of current motif-finding software. We did not impose a constraint that the motif must be an inverted repeat sequence on the search. This yielded a 20-nt motif present in 40 Zur-binding sites and matching the previously reported Zur box consensus (Figure 2A and B) [25]. The conserved nucleotides at positions 4, 5, 15 and 16 corresponded to the most critical for Zur binding (Figure 2C) [11].

**Figure 3:**
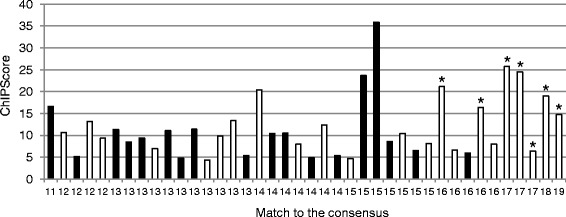
**Relationship between Zur binding** ***in vivo*** **and similarity to the Zur box consensus.** The ChIPScore is scored against the number of nucleotides matching the 20 nt-consensus sequence. Zur box motifs located in intragenic regions (black bars) and Zur box motifs located in intergenic regions (white bars) are indicated. Stars indicate the Zur box motifs belonging to the Zur regulon as previously characterized [10].

**Figure 4:**
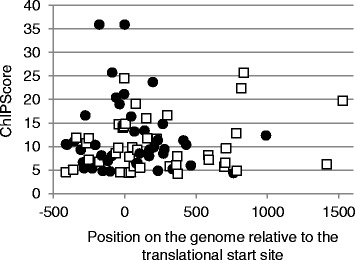
**Relationship between Zur binding** ***in vivo*** **and the position of the detected Zur-binding sites.** The ChIPScore is plotted against the position of the Zur binding sites on the chromosome relative to the closest translational start sites. Black circles, Zur-binding sites in a region containing a predicted Zur box motif; white squares, Zur-binding sites without significant match to the Zur box consensus.

Seven predicted Zur boxes occurred in the promoter regions belonging to the Zur regulon, validating our *in silico* approach. The 33 newly identified Zur boxes were associated to *in vivo* Zur binding in inter- as well as in intragenic regions with a broad distribution of ChIPScores (see Methods), which reflect the strength of *in vivo* Zur-DNA interaction (Additional file 3: Table S2). Correlation between ChIPScores and the degree of similarity of each Zur box to the consensus sequence was investigated. As shown in Figure 3, no clear correlation was observed. It is possible that differences in ChIPScores result not only from Zur-DNA binding affinity but also from differences in cross-linking efficiency at some DNA regions. Interestingly, the highest ChIPScores (>15) were associated to Zur boxes located less than 50 bp from a translational start site (Figure 4) suggesting that the genomic location could contribute to the strength of Zur binding *in vivo*.

Next, the Zur box consensus was submitted to FIMO [31] to identify putative Zur box in all the *B. subtilis* genome. Hits with a *p* value of ≤ 10^−6^ were regarded as significant, resulting in 167 candidates including the 40 Zur boxes associated with *in vivo* Zur binding (see above) (Additional file 4: Table S3). These results showed that only a set of potential Zur boxes were bound by Zur, at least in the conditions used.

Half of the Zur-binding sites detected by ChIP-on-chip did not display a significant match to the Zur box consensus. As shown in Figure 4, these sites were located in inter- as well as in intragenic with some high ChIPScores (>15). Using MEME, we were unable to identify a common DNA sequence motif among Zur targets that lack a canonical Zur box motif.

### The intragenic Zur box from *ymaD* promotes a Zur-mediated repression when placed within a promoter region

We further investigated the functionality of an intragenic Zur box to be recognized by the wild-type Zur protein to promote a repressive effect on transcription. We chose the intragenic Zur box located in the *ymaD* gene encoding a putative peroxiredoxin. Peroxiredoxins are important for antioxidant defense by reducing hydrogen peroxide (H_2_O_2_), which induces the zinc uptake system ZosA in *B. subtilis* [19]. A Δ*ymaD* deleted strain (Table 1) was constructed and the sensitivity of this *ymaD* mutant to H_2_O_2_ was tested in liquid medium. After 30 min of growth in the presence of 400 μM H_2_O_2_, 0.005% and 1% survival was observed for the mutant and the wild-type cells, respectively (Additional file 5: Figure S2). Thus, deletion of the *ymaD* gene increased the sensitivity of the cells to H_2_O_2_, indicating that YmaD plays a role in protecting cells against oxidative stress.

**Figure 5:**
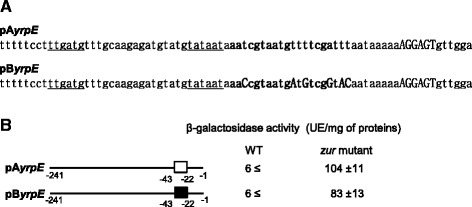
**Structure of the** ***yrpE*** **promoter region and expression of** ***yrpE*** **’-** ***lacZ*** **transcriptional fusions. (A)** Detailed view of the *yrpE* promoter region in the two transcriptional fusions. The σ^A^-dependent recognition site is underlined, the ribosome-binding site is capitalized. The Zur box motif is bold. In pA*yrpE*, the Zur box motif is the native sequence. In pB*yrpE*, the Zur box motif is the intragenic Zur box sequence from *ymaD*. The six nucleotides differences are indicated in bold capital letters. **(B)** Expression of the transcriptional pA*yrpE*’-*lacZ* and pB*yrpE*’-*lacZ* fusions in the wild-type (WT) strain and the Δ*zur* deleted mutant. Nucleotides are numbered relative to the translational start site of the *yrpE* gene. Location of the Zur motif is indicated by boxes. White box, native Zur box sequence from the *yrpE* promoter region; black box, Zur box sequence identified within the *ymaD* gene. β-galactosidase activities were measured at least 3 times independently.

Subsequently, we tested the ability of the *ymaD* intragenic Zur box to promote Zur-mediated regulation. In a previous *in silico* prediction, a unique Zur box motif was detected in the *yrpE* promoter region from nucleotide −22 to −41 relative to the translational start site [25]. *yrpE* is transcribed from a σ^A^-dependent promoter (Figure 5A) [26]. Two types of transcriptional fusions with the promoter region of the *yrpE* gene were constructed. First a pA*yrpE*'-*lacZ* fusion was obtained using the *yrpE* promoter sequence spanning from −241 to −1 related to the translational start site, which included the native Zur box sequence. The fusion sequence was integrated at the *amyE* locus of the wild-type and Δ*zur* strains (Table 1). Expression of the pA*yrpE* promoter was 20-fold increased in a Δ*zur* mutant compared to the wild-type (Figure 5B). In the second pB*yrpE*'-*lacZ* fusion, the native Zur box of *yrpE* was replaced by the intragenic Zur box sequence from *ymaD* (Figure 5A). This fusion exhibited 16-fold higher level in Δ*zur* cells than in the wild-type (Figure 5B). Thus, the intragenic Zur box sequence from *ymaD* was recognized by Zur to mediate transcriptional repression when located in a promoter region. This confirmed the hypothesis that intragenic Zur box sequences are functional Zur binding sites.

### Intragenic Zur boxes: a role in transcription elongation?

**Figure 6:**
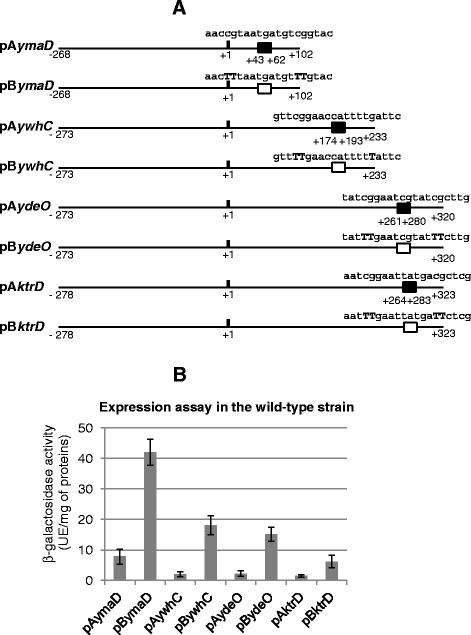
**Expression of various transcriptional fusions in the wild-type and the Δ** ***zur*** **strains. (A)** Scheme of the DNA regions inserted upstream from the *lacZ* reporter gene. Nucleotides are numbered relative to the translational start site of the *ymaD*, *ywhC*, *ydeO* and *ktrD* genes. The native intragenic Zur boxes are indicated by black boxes. The mutated intragenic Zur boxes are indicated by white boxes. The generated point-mutations in the Zur box sequences are indicated in bold capital letters. **(B)** Cells were grown in LB medium. β-galactosidase activities were determined in extracts prepared from exponentially growing cells at OD_600_ of 0.6.

Some transcription factors, such as *B. subtilis* CodY and CcpA regulators, can regulate the transcription elongation by a roadblock mechanism [32,33]. Thus, we tested whether several intragenic Zur binding sites could participate in such a regulatory process. The *ydeO*, *ywhC* and *ktrD* genes contain intragenic Zur-binding sites and encode putative membrane protein, potential zinc metalloprotease, and K^+^-transporting ATPase, respectively. They are transcribed from σ^A^-dependent promoters [26]. Different DNA regions were fused to the *lacZ* reporter gene and integrated at the *amyE* locus of the wild-type strain (Figure 6A, Table 1). The pA*ymaD*, pA*ydeO*, pA*ywhC* and pA*ktrD* fusions contained the promoter region and the start of the encoding-sequence including the native Zur box. The pB*ymaD*, pB*ydeO*, pB*ywhC* and pB*ktrD* fusions contained symmetric mutations in each half-site of the Zur box at position 4 and 5 (Figure 6A) because these point- mutations were shown to completely abrogate Zur binding (Gabriel et al., [11]). β-galactosidase activities were examined during exponential growth (Figure 6B). Expression values of the pA*ymaD*, pA*ydeO*, pA*ywhC* and pA*ktrD* fusions ranged between 2 and 42 units of β-galactosidase activity. In contrast, the pB*ymaD*, pB*ydeO*, pB*ywhC* and pB*ktrD* fusions were 4- to 9-fold upregulated (Figure 6B) suggesting that intragenic Zur boxes mediate a transcriptional *cis*-repressive effect. Expression of the pA and pB fusions was also tested in a *zur* mutant but similar levels of expression was observed in Δ*zur* and wild-type cells (data not shown).

### Zur-binding sites overlap genes responding to disulfide stress

Our analyses revealed that a large overlap existed between the location of Zur-binding sites and genes whose expression responds to disulfide stress [24]. Zur-binding sites were located less than 400 bp upstream of the translational start site or in the coding sequences of 31-activated and 19-repressed genes in response to diamide stress (Additional file 6: Table S4). Furthermore, Zur-binding sites are associated to 10 genes (*citR*, *cysK*, *ilvA*, *katA*, *S1408*, *pps*, *ybxG*, *ymaD*, *yusD*, *yvcI*) reported to be directly regulated by Spx [24], suggesting that Zur- and Spx-mediated regulations partially overlap.

**Figure 7:**
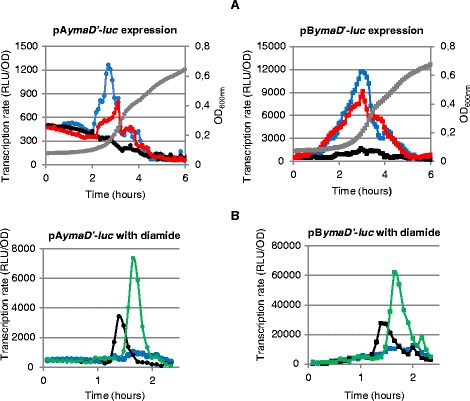
**Expression of** ***ymaD*** **in various genetic backgrounds and after diamide treatment. (A)** Expression of pA*ymaD* and pB*ymaD* fusions in the wild-type (WT) strain (blue lines), in Δ*zur* (red lines) or Δ*spx* cells (black lines). Growth was monitored by measuring the optical density at 600 nm: grey line, wild-type. The Δ*zur* and Δ*spx* stains grew with the same growth profile than the wild-type. **(B)** Promoter activity of pA*ymaD* and pB*ymaD* without diamide treatment (blue lines), with 0.1 mM diamide (black lines) or with 0.5 mM diamide (green lines). Final concentration of 0.1 or 0.5 mM diamide was added at t = 1 hour when cells reached an OD_600_ of 0.2 in LB medium. For each strain, one representative curve, out of three independent replicates realized, is shown.

To investigate a potential role of an intragenic Zur box to mediate a disulphide stress response, we choose to test the effect of diamide treatment on *ymaD* expression in wild-type and Δ*spx* genetic backgrounds. As it is known that LacZ activity is very sensitive to diamide addition, we constructed a pA*ymaD*'-*luc* fusion between the region from nucleotide −268 to +102 relative to *ymaD* translational start site and the luciferase gene. A second pB*ymaD*'-*luc* fusion was constructed containing point-mutations in the intragenic Zur box. Luciferase activity was recorded during the growth in LB medium. Expression of pB*ymaD*'-*luc* was 10-fold higher than expression of pA*ymaD*'-*luc* (Figure 7A) confirming the results observed with the *lacZ* fusions (see above).

No expression of pA*ymaD*'-*luc* and pB*ymaD*'-*luc* was detected in a Δ*spx* mutant (Figure 7A), in keeping with the Spx-dependent activation of *ymaD* [24]. In the wild-type cells, 0.1 or 0.5 mM diamide treatment reproducibly increased pA*ymaD*'-*luc* expression (Figure 7B). This indicated that Zur binding to the Zur box of pA*ymaD* did not interfere woth Spx activity. The same pattern of induction was observed with pB*ymaD* (Figure 7B). Thus, the intragenic Zur box did not appear involved in the Spx-dependent upregulation.

## Discussion

Using the ChIP-on-chip methodology, we identified 80 enriched DNA-regions in the *B. subtilis* chromosome that are reproducibly bound by the Zur repressor under abundant zinc growth conditions. We recovered the known Zur regulon and confirmed that the predicted Zur boxes present upstream of *yrpE* and *rpmGC* [17,25] are functional binding sites *in vivo*. These data allow to define the Zur primary regulon, which is now composed of 7 transcription units. Consequently, the whole Zur binding sites identified by ChIP-on-chip appear relevant. Remarkably, a second set of 33 newly identified sites bound by Zur contained a Zur box motif, which differs from the Fur and PerR boxes at positions 4, 5, 15, and 16 and displays conservation of bases at flanking positions that are not strongly conserved in Fur and PerR boxes (positions 1,2,3 and 18, 19, 20) (Figure 2) [11,29]. The last set of Zur binding DNA sites did not contain any direct-repeat sequence or any common motif by the standard bioinformatic methods that we used, suggesting that Zur recognizes degenerated Zur box sequences or that other factors are required for Zur binding at these sites. Interestingly, the Zur binding sites with and without predicted Zur box exhibit a similar distribution relative to coding sequences, with an enrichment around the translation start site (Figure 4), suggesting that both types of sites are functionally similar.

A surprising result from this study was the finding that 35 Zur binding sites are located in intragenic regions. Sixteen of those contain a predicted Zur box. This contrasts sharply with *in silico* studies, which tend to consider transcription factor-targets within coding sequences as artefacts. Analysis with transcriptional fusions using the *lacZ* reporter gene allowed us to show that the intragenic Zur box sequence from the *ymaD* gene was fully functional for Zur-mediated repression when placed in a promoter region. Thus, the experimental strategies used in this study revealed unexpected and functional Zur binding sites. In addition, we showed that the intragenic Zur box within *ymaD*, *ydeO*, *ywhC* and *ktrD* genes had a *cis*-repressive effect (3- to 10-fold) on transcription. Expression of these genes was also tested in a Δ*zur* mutant but similar levels of expression was observed in Δ*zur* and wild-type cells (data not shown). As disruption of zinc homeostasis in Δ*zur* cells likely affects many cellular processes, the direct role of Zur binding to the intragenic Zur boxes could be masked by other regulatory effects.

Our study highlights the presence of Zur boxes within a subset of genes encoding functions related to metal ion homeostasis or oxidative stress. We showed that the *ymaD* gene codes for putative peroxiredoxin-related protein, which plays a role in protecting cells against oxidative stress. Interestingly, *ymaD* is also under direct regulation by Spx [24] a global transcription key regulator for maintaining redox homeostasis of *B. subtilis* cells exposed to disulfide stress [34]. The intragenic Zur box of *ymaD* did not appear to play a direct role on the Spx-dependent regulation. Remarkably, we observed that the *ymaD* transient induction upon exposure to diamide was abolished in Δ*zur* cells (data not shown) pointing to an interconnection of the Zur- and Spx-mediated responses. As the degradation of Spx is ensured by the ClpXP protease [35], disruption of zinc homeostasis in Δ*zur* cells could impact on the activity of the Zn-dependent protein ClpX and, as a consequence, on the turnover of Spx. Overall, it was not possible to conclude about the direct role of Zur to mediate a regulation via the intragenic Zur boxes because of Δ*zur* deletion may entail significantly changes in other transcription factors activity. In this intricate regulatory network, the binding of Zur to intragenic Zur boxes may contribute to fine-tune gene expression in response to zinc availability.

The interconnection between Zur and Spx may involve more than one gene as Zur binding sites were detected within or near 10 Spx-regulated genes and overall near 50 genes responsive to diamide stress (Additional file 6: Table S4). Overlap between disulfide and oxidative stress responses was previously identified in *B. subtilis* for the *katA* gene encoding a catalase. Expression of *katA* is under the dual control of PerR [29] and Spx [24] regulators. In addition, a regulatory interplay between the responses to zinc deprivation and disulfide stress has been described in *Streptomyces coelicolor*, where the activity of the thiol-disulfide metabolism regulator σ^R^ is induced upon zinc limitation [36]. Our results emphasize the complex interplay between the regulatory networks controlling zinc homeostasis and redox homeostasis, especially the oxidative and disulfide stress responses.

## Conclusions

The Chip-on-chip approach used in this study allowed to considerably expand the catalogue of *in vivo* Zur-binding sites to 80 inter- as well as intragenic regions. Half of those is associated with an *in silico* predicted Zur box. The binding of Zur to the newly identified targets may contribute to fine-tune gene expression under specific conditions, our results highlighting a complex link between Zur and the disulfide stress response. Intragenic Zur boxes could be involved in an intricate mechanisms of regulation of the transcription elongation, possibly with other transcriptional factors. Future investigations will be required to investigate the role of Zur binding sites in transcriptional regulation.

## Methods

### Bacterial strains and growth conditions

The *B. subtilis* strains used in this work are listed in Table 1. *E. coli* and *B. subtilis* cells were grown in Luria-Bertani (LB) medium or in MS medium containing 62 mM K_2_HPO_4_, 44 mM KH_2_PO_4_, 17 mM trisodium citrate, 11 mM K_2_SO_4_, 0.4% glucose, 0.06% L-glutamine, 0.01% L-tryptophan, 0.1% casamino acids, 1 mM MgSO_4_, 1 mM CaCl_2_, 100 μM FeCl_3_ citrate, 112 μM ZnCl_2_; 5 μM MnCl_2_; 2.5 μM CuCl_2_. Antibiotics were added at the following concentrations when required: 100 μg ampicillin ml^−1^; 5 μg kanamycin ml^−1^; 10 μg erythromycin ml^−1^; 5 μg chloramphenicol ml^−1^; 60 μg spectinomycin ml^−1^. Solid media were prepared by addition of 20 g Agar noble l^−1^ (Difco). Standard procedures were used to transform *E. coli* [37] and *B. subtilis* [38].

### DNA manipulations

DNA manipulations and cloning procedures were performed as described elsewhere [37] according to standard procedures. Restriction enzymes, *Pfu* DNA polymerase and phage T4 DNA ligase were used as recommended by the manufacturer (Biolabs). DNA fragments were purified from agarose gels using the QIAquick kit (Qiagen).

### Construction of plasmids and strains

A *B. subtilis* strain expressing a C-terminal SPA-tagged Zur protein (hereafter Zur^SPA^) was constructed by chromosomal integration of a translational fusion between the *zur* coding sequence and the sequential peptide affinity (SPA) tag sequence [39,40], resulting in the BSAS36 strain expressing Zur^SPA^ under the control of its native promoter as unique source of Zur. In this purpose, the *zur* coding sequence (from nucleotide +13 to + 435 relative to the translational start site) was amplified by PCR with oligonucleotides creating an *Acc*651 restriction site at the 5′ end (5′-GGAATTGGTACCgaagcgctgaacctattaaaa-3′) and a *Nco*I restriction site at the 3′ end of the fragment (5′-GGAATTCCATGGcgcagtagtgttttcttggtt-3′). The PCR product was cloned into plasmid pMUTIN-SPA subsequent to digestion with *Acc*651 and *Nco*I [41]. The resulting plasmid was used to transform *B. subtilis* and to select for erythromycin-resistance. Integration was confirmed by PCR and verified by DNA sequencing.

The *zur* mutant BSAS45 was constructed by homologous replacement of the Zur coding sequence with the kanamycin-resistance gene *aphA3* using a joining PCR technique [42]. The *aphA3* gene was first amplified. The region upstream of the *zur* gene (nucleotides −887 to +65 relative to the translational start site) was amplified by PCR with a 21 bp *aphA3* fragment at its 3′ end. The region downstream of *zur* (nucleotides +366 to +1321) was amplified with a 21 bp *aphA3* fragment at its 5′ end. The three DNA fragments were combined and then a PCR reaction was performed with the two external oligonucleotides. The final product, corresponding to the two regions flanking *zur* with the inserted *aphA3* cassette in between, was purified from a gel and used to transform *B. subtilis*. Integration and deletion were confirmed by PCR and verified by DNA sequencing. The *ymaD* mutant BSAS239 was constructed by the same strategy. The region upstream of the *ymaD* gene (nucleotides −921 to +90 relative to the translational start site) was amplified by PCR with a 21 bp *aphA3* fragment at its 3′ end. The region downstream of *ymaD* (nucleotides +400 to +1431) was amplified with a 21 bp *aphA3* fragment at its 5′ end. The joining PCR, corresponding to the two regions flanking *ymaD* with the inserted *aphA3* cassette in between was used to transform *B. subtilis*.

The *spx* mutant BSAS296 was constructed by homologous replacement of the Spx coding sequence with a spectinomycin-resistance gene *spc*. The *spc* gene was first amplified. The region upstream of the *spx* gene (nucleotides −861 to +57 relative to the translational start site) was amplified by PCR with a 21 bp *spc* fragment at its 3′ end. The region downstream of *spx* (nucleotides +348 to +1270) was amplified with a 21 bp *spc* fragment at its 5′ end. The three DNA fragments were combined and then a PCR reaction was performed with the two external oligonucleotides. The final product, corresponding to the two regions flanking *spx* with the inserted *spc* cassette in between, was purified from a gel and used to transform *B. subtilis*. Integration and deletion were confirmed by PCR and verified by DNA sequencing.

To construct transcriptional fusions with the *lacZ* reporter gene, DNA fragments corresponding to the various promoter regions under investigation were amplified by PCR. Oligonucleotides were used to create an *Eco*RI restriction site at the 5′ end and a *Bam*HI restriction site at the 3′ end of the fragments. PCR products were cloned into plasmid pAC6 subsequent to digestion with *Eco*RI and *Bam*HI [43]. In this way, the promoter region of *yciC* (from nucleotide −312 to −1 relative to the translational start site) was fused with the *lacZ* reporter gene. The transcriptional fusions with the *lacZ* gene were subsequently integrated at the *amyE* locus of *B. subtilis* (Table 1). To generate pB*ymaD*, pB*ydeO*, pB*ywhC* and pB*ktrD* fusions, we used large primers introducing point-mutations in the Zur box motifs (Figure 6A). The resulting constructs were verified by DNA sequencing. β-galactosidase specific activities were measured during exponential phase growth in LB medium, as described by Miller with cell extracts obtained by lysozyme treatment [44]. One unit of β-galactosidase activity was defined as the amount of enzyme that produces 1 nmol *o*-nitrophenol min-1 at 28°C. The mean values and standard deviations of at least three independent experiments are shown.

To construct transcriptional fusions with the *luc* reporter gene, we used the assembly Gibson's procedure [45] to obtain transcriptional fusions with *luc* instead of the *lacZ* gene. The PUC18cm-luc plasmid [46] was used as template to amplify the *luc* reporter gene. The sequence of the resulting constructs were verified by DNA sequencing. The mean values and standard deviations of at least three independent experiments are shown.

### Luciferase assay

For the detection of luciferase activity, strains were first grown in LB medium to an optical density at 600 nm (OD_600_) of 2. Cells were then centrifuged and resuspended in fresh LB medium, adjusting all the cultures to an OD_600_ of 1. These pre-cultures were then diluted 20 fold in fresh LB medium and 200 μl was distributed in each of two wells in a 96-well black plate (Corning). 10 μl of luciferin were added to each well to reach a final concentration of 1.5 mg/ml (4.7 mM). The cultures were incubated at 37°C with agitation in a PerkinElmer Envision 2104 Multilabel Reader equipped with an enhanced sensitivity photomultiplier for luminometry. The temperature of the clear plastic lid was maintained at 38°C to avoid condensation. Relative Luminescence Unit (RLU) and OD_600_ were measured at 5 min intervals.

### Genome-wide determination of the Zur-binding sites by ChIP-on-chip

Chromatin Immnunoprecipitation assays were performed to measure the chromosome-wide DNA-binding profiles of Zur, as described previously [26]. Briefly, strain BSAS36 was cultivated at 37°C until an OD_600_ of 0.6 in LB medium with 1 μg erythromycin ml^−1^. Cells were treated with formaldehyde, cellular DNA was extracted and sonicated, and an antibody against the SPA-tag was used to preferentially purify the DNA regions specifically cross-linked to Zur^SPA^. The immuno-precipitated DNA (IP) and the control whole cell DNA extract (WCE) were labeled with Cy3 and Cy5, respectively, and co-hybridized to the *B. subtilis* Roche-NimbleGen tiled microarrays [47].

### Peak sequence extraction and analysis

Identification of peaks corresponding to chromosomal Zur binding sites was performed as described in [48]. IP/WCE ratios (log2) were corrected for dye bias using Loess regression on the MA plot. The signal was smoothed by two rounds of sliding window averaging (29 probes, around 320 bp). Maxima (or minima) were defined as probes for which the smoothed signal is the highest (or lowest respectively) into the window used for smoothing. Peaks within the same 300 bp window were merged. The peak height was calculated as the log2 ratio difference between the smoothed signal values of the maxima and the adjacent minima. In order to quantify enrichment of Zur-bound DNA regions, the signal was smoothed and a ChipScore was calculated as described by Buescher *et al*. [23]. Briefly, this score is based on the distribution of the peak height values and estimates for each peak its relative distance from the median (ChipScore = [height-median]/[upperquartile-median]). Only the regions associated with a peak scoring ≥ 4 [a threshold determined empirically from ChIP–on-chip experiments with the transcription factor CcpA [23]] in both replicates were considered as putative Zur-binding sites in the subsequent analyses.

### Availability

The data discussed in this publication have been deposited in NCBI's Gene Expression Omnibus and are accessible through GEO Series accession number GSE64671 (http://www.ncbi.nlm.nih.gov/geo/query/acc.cgi?acc=GSE64671).

## Additional files

Additional file 1: Figure S1. (A) Effect of ion starvation on the expression of a *yciC*'-*lacZ* transcriptional fusion in various genetic backgrounds. The BSAS39 (*zur* ^+^) and BSAS44 (*zur*::*zur*-*spa*) strains were cultivated in the MS defined medium until OD_600_ of 1. Samples of 2 ml of the cultures were spread onto solid MS medium containing 20 μg.ml^−1^ X-gal. A drop of 10 μl 100 μM EDTA was deposited at the center of each plate. Blue rings corresponded to expression of the fusion in cells around the inhibition zone of EDTA drops. (B) Expression of *yrpE* under the control of Zur Strains were grown in LB medium. Growth was monitored by measuring the optical density at 600 nm: dark circles, wild-type; grey circles, Δ*zur*. Promoter activity of P*yrpE*'-*lacZ* was measured in wild-type (blue circles) and Δ*zur* (red circles) cells. Additional file 2: Table S1. Mapping of Zur DNA binding sites by ChIP-on-chip. Additional file 3: Table S2. Zur box-like motifs located in Zur-binding regions detected by ChIP-on-chip. Additional file 4: Table S3. FIMO analysis of the occurrence of the Zur box motif in the whole *B. subtilis* genome. Additional file 5: Figure S2. Comparison of the effect of hydrogen peroxide on the survival of ΔymaD and wild-type cells. Survival of BSAS239 Δ*ymaD* (open triangles) and wild-type (black triangles) growing cells in LB medium were calculated after 15 and 30 min of challenge with 400 μM H_2_O_2_. Hydrogen peroxide was added at OD_600_ of 0.6. One hundred per cent corresponds to the number of c.f.u. before H_2_O_2_ was applied (approx 5.10^7^ c.f.u. ml^−1^ of each culture). Additional file 6: Table S4. Overlap between Zur binding-sites and response to diamide stress.
