# Supplementary figures and images for: Genome-wide identification of Bacillus subtilis Zur-binding sites associated with a Zur box expands its known regulatory network
Source: BMC Microbiol. 2015 Feb 4;15(1):13. doi: 10.1186/s12866-015-0345-4 (PMC4324032; doi:10.1186/s12866-015-0345-4)

**Figure S1**

**A**

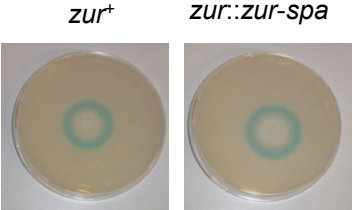

**B**

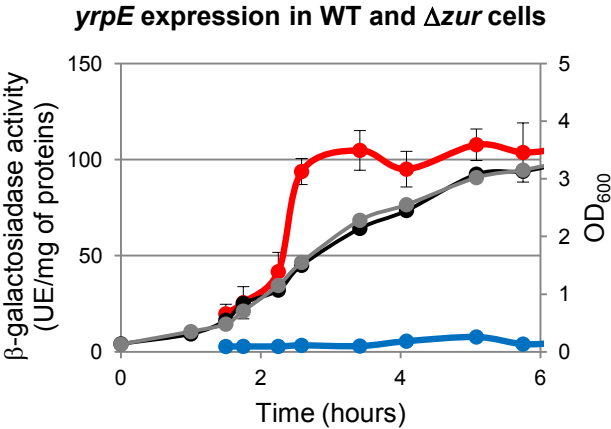

Supplement: Additional file 1: Figure S1. — (A) Effect of ion starvation on the expression of a yciC'-lacZ transcriptional fusion in various genetic backgrounds. The BSAS39 (zur +) and BSAS44 (zur::zur-spa) strains were cultivated in the MS defined medium until OD600 of 1. Samples of 2 ml of the cultures were spread onto solid MS medium containing 20 μg.ml−1 X-gal. A drop of 10 μl 100 μM EDTA was deposited at the center of each plate. Blue rings corresponded to expression of the fusion in cells around the inhibition zone of EDTA drops. (B) Expression of yrpE under the control of Zur Strains were grown in LB medium. Growth was monitored by measuring the optical density at 600 nm: dark circles, wild-type; grey circles, Δzur. Promoter activity of PyrpE'-lacZ was measured in wild-type (blue circles) and Δzur (red circles) cells. [file 12866_2015_345_MOESM1_ESM.pdf]

**Figure S2**

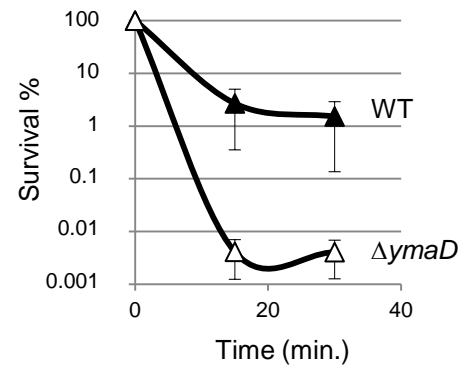

Supplement: Additional file 5: Figure S2. — Comparison of the effect of hydrogen peroxide on the survival of ΔymaD and wild-type cells. Survival of BSAS239 ΔymaD (open triangles) and wild-type (black triangles) growing cells in LB medium were calculated after 15 and 30 min of challenge with 400 μM H2O2. Hydrogen peroxide was added at OD600 of 0.6. One hundred per cent corresponds to the number of c.f.u. before H2O2 was applied (approx 5.107 c.f.u. ml−1 of each culture). [file 12866_2015_345_MOESM5_ESM.pdf]
